# ECG Monitoring Based on Dynamic Compressed Sensing of Multi-Lead Signals

**DOI:** 10.3390/s21217003

**Published:** 2021-10-22

**Authors:** Pasquale Daponte, Luca De Vito, Grazia Iadarola, Francesco Picariello

**Affiliations:** Department of Engineering, University of Sannio, Corso Garibaldi, 107, 82100 Benevento, Italy; daponte@unisannio.it (P.D.); grazia.iadarola@unisannio.it (G.I.); fpicariello@unisannio.it (F.P.)

**Keywords:** electrocardiogram, Compressed Sensing, multiple measurement vector reconstruction, signal recovery, biomedical measurement system, wearable devices, Internet of Things

## Abstract

This paper presents an innovative method for multiple lead electrocardiogram (ECG) monitoring based on Compressed Sensing (CS). The proposed method extends to multiple leads signals, a dynamic Compressed Sensing method, that were previously developed on a single lead. The dynamic sensing method makes use of a sensing matrix in which its elements are dynamically obtained from the signal to be compressed. In this method, for the application to multiple leads, it is proposed to use a single sensing matrix for which its elements are obtained from a combination of multiple leads. The proposed method is evaluated on a wide set of signals and acquired on healthy subjects and on subjects affected by different pathologies, such as myocardial infarction, cardiomyopathy, and bundle branch block. The experimental results demonstrated that the proposed method can be adopted for a Compression Ratio (CR) up to 10, without compromising signal quality. In particular, for CR= 10, it exhibits a percentage of root-mean-squared difference average among a wide set of ECG signals lower than 3%.

## 1. Introduction

Electrocardiogram (ECG) has always been among the most investigated signals due to multiple reasons. ECG monitoring allows preventing cardiovascular and many correlated diseases (such as diabetes and hypertension) [1], and it is widely adopted in surgical interventions, sport activity monitoring, and daily home healthcare [2]. As stated in [3,4], the standard 12-lead ECG recording is able to effectively reflect rich spatial information of the heart’s movements allowing the recognition of specific pathological features [5]; thus, it is widely used in clinic and hospital applications. In a standard 12-lead monitoring system, heart activity is detected by employing up to 10 electrodes placed in standardized positions, which provide information regarding the following: three bipolar limb leads I, II, and III; three augmented limb leads, aVR, aVL, and aVF; and six precordial leads, V1, V2, V3, V4, V5, and V6. The cardiologist is able to make a diagnosis by comparing the ECG signals acquired from the different leads with reference ECG waveforms reported in the scientific literature. The minimum number and set of selected leads depend on the need of observing specific waveforms that could be then related to specific pathologies. As a consequence, an analysis aimed at determining the minimum number of sensors to be used, as reported in [6], cannot be carried out unless referring to specific pathologies. Although providing complete ECG information, the acquisition of 12-lead ECG in people’s daily lives is still a challenge [3]. In fact, several devices are occasionally used for at-home patient monitoring; nevertheless, they are still not convenient and comfortable enough for 24/7 usage due to the obtrusive 12-lead setting [3].

Nowadays, continuous remote monitoring of physiological parameters is demanded for patients performing therapy at home, and it is currently allowed by Internet-of-Things technology, which in this case takes the term of Internet of Medical Things (IoMT). IoMT generally constitutes an elaborated paradigm, with medical things meaning wearable devices and smart sensors tied to human bodies (sometimes implanted in bodies), allowing the acquisition of biosignals and other vital parameters. Standard configurations of IoMT remote monitoring systems, where ECG signal is acquired at Nyquist rate, do not suit storage and transmission requirements as a huge amount of data need to be stored and transmitted [7], especially when a high number of patients is monitored [8]. Moreover, power consumption is often related to signal data rate, since data transmission is the main cause of energy dissipation in many interfaces (such as Wireless Local Area Network (WLAN) and Wireless Wide Area Network (WWAN) interfaces) [9].

In Wavelet or Fourier domains, ECG signals are demonstrated to be sparse [9], i.e., they can be represented by a reduced number of samples. Relying on sparsity property, the technique of Compressed Sensing (CS) has been proposed in order to reduce the number of samples representing the signals of interest and to reconstruct their digital version without compromising signal quality [10]. The CS has been vastly investigated in the field of biomedical signal processing not only for ECG monitoring but also for electroencephalogram, electromyogram, electrooculogram, galvanic skin response, and heart sound signals [9,11,12]. Adopting CS entails a reduction in data rate with respect to signal information content. Among the resulting benefits, it is possible to mention not only an increase in battery lifespan of sensor nodes but also an easier allocation of time slots for communications, e.g., when multiple systems are used sharing the same band or different physiological signals are acquired simultaneously.

ECG acquisition based on CS has been implemented both in hardware and software by means of analog or digital methods. The main advantage of analog methods, typically known as compressive sampling, consists in decreasing the sampling rate by employing architectures working below the Nyquist limit [13]. On the other hand, digital methods are not intended to sample under the Nyquist rate but are intended to reduce the number of samples to be transmitted. Digital approaches have the main advantage of lower power consumption due to the lack of dissipating devices (such as mixers) in hardware implementations; thus, they are considered preferable in wireless applications [9]. In reality, other compression algorithms for ECG signals are built on transforms in Fourier or Wavelet domains [14,15,16,17,18] or on fractal-based transforms [19]. These algorithms are potentially able to reconstruct the original ECG signal with good performance. Nevertheless, their high computational complexity and buffer requirements do not adapt to real time implementations of ECG monitoring [14,15]. Differently from the aforementioned compression algorithms, the CS reduces instead the computational complexity related to the compression phase such that it complies with the limited physical resources of IoMT sensor nodes. Instead, the most computationally heavy task which consists of the waveform reconstruction relies in the higher layers of the IoMT architecture that are usually deployed in the cloud where powerful resources are available. Research is also in progress in order to perform anomaly detection directly from compressed samples without the need of reconstructing the waveforms [20].

Although multiple electrodes and leads are actually adopted by measurement systems in biomedical field, ECG monitoring through CS has been addressed in the literature mainly in the case of one electrode with one lead [9,13,21,22,23,24,25,26]. The aim of this paper is to propose a digital CS-based method for ECG monitoring of multi-lead signals. Obviously, a significant part of the information content sensed by various electrodes is common to all the signals on the different leads. Such property is exploited by the proposed method by jointly reconstructing the multi-lead signals from the same support. The multi-lead proposed method implements a dynamic deterministic approach [26] in the sense that the compression mechanism is adapted to the acquired signals to include more information on cardiac features and improve reconstruction quality. The method is designed to be implemented in the Ambient-Intelligent Tele-monitoring and Telemetry for Incepting and Catering Over hUman Sustainability (ATTICUS) system described in [27]. This system consists of a smart T-shirt, called S-WEAR, capable of monitoring from one up to six ECG leads. S-WEAR has a modular architecture which can be adapted for multi-leads monitoring without substantially increasing overall costs of the entire system and without compromising its comfort. The ATTICUS system is characterized by a three-level Decision Support System (DSS). The first level is installed in the S-WEAR and allows automatically detecting a limited set of anomalies, such as when the heart rate is too high. The second level is installed on an electronic device, called S-BOX, that is installed at the patient’s home, and is able to detect a broader range of anomalies, such as irregular heartbeat. Finally, the third level is installed on the server, and it makes use of advanced machine-learning techniques to decide if it is necessary to notify the alarm of the physician at the monitoring center. They allow automatic detection: atrial fibrillation, ventricular tachycardia, congestive heart failure, and four types of arrhythmia conditions. Some of these machine learning techniques work on multi-lead acquisitions for assuring high accuracy [27]. If an alarm is sent to the physician, long recordings of the acquired multi-lead signals before and after the detected event are sent to her/him in order to make a diagnosis. This requires the storage of a large amount of data that need to be compressed.

A preliminary version of the method has been introduced in [28], where the multi-lead reconstruction was carried out by dynamically evaluating the signal frame acquired on the first lead through a threshold set by a percentile. In the work presented here, the following innovations have been added: (i) the dynamic sensing matrix is built from a combination of the most significant leads in terms of information content, i.e., lead II and aVF, instead of the first lead; (ii) for the reconstruction, a comparison in terms of signal quality and execution time between Multiple Sparse Bayesian Learning (M-SBL) and the Multiple FOCal Underdetermined System Solver (M-FOCUSS) is performed; (iii) experimental validation is extended with a set of signals of subjects affected by specific pathologies; and (iv) an experimental comparison with other four relevant literature methods proposing CS for multi-lead ECG monitoring has been added.

The rest of the paper is organized as follows. An overview of the CS-based methods available in the literature for the compression of multi-lead ECG signals is proposed in Section 2. Section 3 presents the proposed method by detailing the two phases of compression and reconstruction. In Section 4, the implementation of the proposed method is described. Section 5 illustrates the experimental results of the proposed method for several sets of signals. Specifically, in Section 5.1, an analysis versus the regularization parameter used in the reconstruction algorithm is presented. In Section 5.2, the performance of the proposed method versus the compression ratio and the number of leads is analyzed. In Section 5.3, an experimental comparison of the results of the proposed method with those achieved other four relevant literature methods is reported. Lastly, Section 6 is devoted to conclusions.

## 2. Related Works

As stated above, in the literature, few studies are focused on the use of CS methods for multi-lead monitoring [7,29,30,31]. Those methods outperform the single lead CS methods because they rely on the fact that the ECG signals from multi-lead channels are not independent but they have the electrical heart vector as a common source of information. In particular, the ECG signals on the different leads correspond to the projections in different directions of the electrical heart vector.

In [31], the adopted multi-lead CS method is based on Filtered Modulated-Multiplexer (FM-Mux) architecture. In this case, firstly, each ECG signal is modulated with a pseudo-random sequence having a rate higher than the analyzed bandwidth. Secondly, the signals are convoluted with low-pass linear time-invariant (LTI) filters having a bandwidth equal to the rate of the sequence. Finally, the signals are added together onto a single channel and uniformly sampled by an Analog-to-Digital Converter (ADC) at a rate lower than the Nyquist one and equal to the rate of the pseudo-random sequence. The results of [31] demonstrated that the reconstruction quality in terms of PRD is lower than 9% by considering a Compression Ratio (CR) of 5.

In [30], the ECG signals are acquired by ADCs working at the Nyquist rate and then compressed by multiplying the acquired samples with a sparse binary matrix. The results demonstrate that the adopted method achieves good reconstruction quality, i.e., PRD lower than 9%, for CR around 3.

A multi-lead CS method, based on the same sparse binary matrix as before is proposed in [7]. In this case, the performance of several Weighted mixed-norm minimization (WMNM)-based joint sparse recovery algorithms was assessed, and a PRD around 7% was achieved with a CR of 7 by means of the Prior Weighted MNM (PWMNM) algorithm.

In [29], a sparse binary matrix was adopted as a sensing matrix together with a sparsity matrix based on the Daubechies 6 wavelet. In this case, a PRD lower than 9% was achieved with a CR around 4 [29].

The above-mentioned methods are based on sensing matrices that are randomly built; on the other hand, in the work here presented, a deterministic matrix is adopted. This deterministic matrix was already tested in [26] for single lead monitoring. In this paper, its use is analyzed in the case of multi-lead monitoring with the aim of outperforming the performance in terms of reconstruction quality of random-based approaches.

## 3. The Proposed Method

IoMT networks allow constantly assessing the health status of subjects monitored through biomedical measurement systems [26]. The working principle of a generic IoMT-enabled system is based on the transfer of information from several sensor nodes to a cloud server that constitute a physical layer and the information integration layer of the IoMT model [32], respectively. Each sensor node acquires and transmits the biosignal samples. The cloud server stores the received samples, which are later employed to recover original information about the health status. Therefore, in an IoMT network—which can be shared also among thousands of nodes—considerable data rates have to be handled.

Generically, the sensor node of the ECG monitoring system consists of multiple electrodes that form *L* leads. The ECG signal on each lead $x_{l}\left(t\right)$, with l=1,2,⋯,L, is acquired through an analog front-end and an Analog-to-Digital Converter (ADC) working at the Nyquist rate. A record of *N* acquired samples is here represented as the vector $x_{l}$. Overall, in the time frame of each record, the ECG monitoring system acquires and transmits a matrix of N×L samples.

(1) $$X=\left[x_{1},x_{2},\cdots ,x_{L}\right].$$

Let each vector acquired at the Nyquist rate, i.e., each column $x_{l}$ of the matrix X, be sparse, i.e., represented by a few non-null coefficients in a given transform domain described by a *sparsity matrix* $\Psi \in R^{N\times N}$. In this case, each vector $x_{l}$ can be expressed as follows:

(2) $$x_{l}=\Psi c_{l},$$

where $c_{l}$ is a vector of the signal coefficients with few nonzero elements. Overal, the matrix X can be expressed as follows:

(3) X=ΨC,

with $C=\left[c_{1},c_{2},\cdots ,c_{L}\right]$.

The ECG signal is usually sparse in several Wavelet and Fourier domains. As reported in [9], one of the sparsity matrices achieving the highest reconstruction performance is obtained by means of the Mexican hat wavelet kernel. In this paper, a Mexican hat wavelet matrix $\Psi^{\prime }$ defined according to [33] is used:

(4) $$\Psi^{\prime }=\left[\psi \left(2,0\right),\psi \left(2,2\right),\ldots ,\psi \left(2,2\left\lfloor \frac{N-1}{2}\right\rfloor \right),\psi \left(4,0\right),\psi \left(4,4\right),\ldots ,\psi \left(4,4\left\lfloor \frac{N-1}{4}\right\rfloor \right),\ldots ,\psi \left(N,0\right)\right],$$

where $\psi \left(a,b\right)\in R^{N\times 1}$ is a vector that describes the Mexican hat wavelet function having $a=2^{m}$, and $m=\{1,\cdots ,\left\lfloor \log_{2}\left(N\right)\right\rfloor \}$ and $b=\left\{0,a,2a,\cdots ,a\left\lfloor \frac{N-1}{a}\right\rfloor \right\}$ denote scale and translation parameters, respectively:

(5) $$\psi \left(a,b\right)=\frac{2}{\sqrt{3a}\;\cdot \;\pi^{1/4}}\;\cdot \;\left[1-{\left(\frac{n-b}{a}\right)}^{2}\right]\;\cdot \;e^{-\frac{1}{2}{\left(\frac{n-b}{a}\right)}^{2}},$$

with $n={\left[0,\cdots ,N-1\right]}^{T}$.

**Figure 1:**
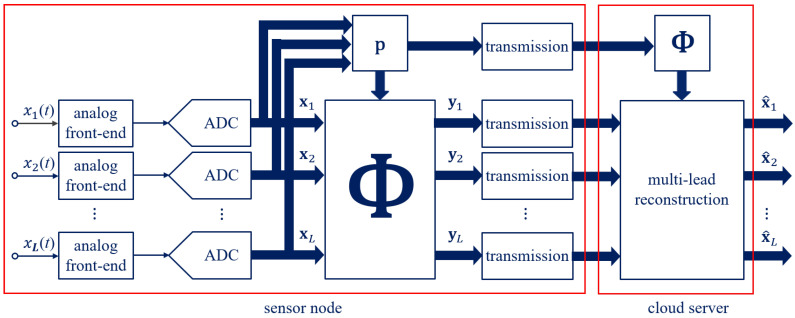
Multi-lead method based on dynamic CS.

The multi-lead method proposed in this paper for ECG monitoring is intended to optimize, through CS [10], the transfer of information in IoMT networks. In particular, as depicted in Figure 1, the sensor node sub-samples the multi-lead ECG signals coming from electrodes, while the cloud server jointly reconstructs them. The sensor node can be implemented by simple hardware consisting of electrodes and leads along with a microcontroller and a radio frequency interface, since the ECG signals are firstly acquired at the Nyquist rate and then compressed. The joint reconstruction is instead relied on by the cloud server and is typically characterized by unconstrained resources. The multi-lead proposed method consisting in the two phases of compression and reconstruction is detailed in the following.

In the sensor node, the vector acquired on each lead, $x_{l}$, is compressed in an *M*-dimensional measurement space, with M<N. The core idea behind the proposed method is the formulation of a dynamic *sensing matrix* Φ of size M×N that sub-samples every frame of *N* samples of the multi-lead signals and that is effective for a joint reconstruction. In particular, data reduction in sub-sampling systems is typically expressed by the *Compression Ratio* (CR).

(6) $$\mathrm{CR}=\frac{N}{M}.$$

In order to build the dynamic sensing matrix Φ, a vector p of *N* binary digits must be preliminarily introduced. The vector p is defined from a vector $x_{0}\in R^{N}$ that constitutes a proper combination of the leads with the most significant contribution. Specifically, the vector $x_{0}$ is built as the root mean square of the bipolar limb lead II and the augmented limb lead aVF.

(7) $$x_{0}=\sqrt{\frac{1}{2}\left(x_{II}^{2}+x_{aVF}^{2}\right)}.$$

Actually, different lead combinations can also be considered. For example, another combination allowing to sub-sample with high CR values is the root mean square of the leads II, aVR and V6, where one lead for each lead group is selected. Generally, lead II is the most commonly used one for accurately assessing cardiac rhythm as it usually provides a good view of the P wave [34], while including precordial leads may help to follow R wave progression. Another vector, called $x_{p}$, is obtained from the vector $x_{0}$, and the average $x_{avg}$ of $x_{0}$ is defined as follows.

(8) $$x_{p}\;=\;|x_{0}-x_{avg}|.$$

Then, a threshold value $x_{th}$ is determined starting from the vector $x_{p}$. The threshold $x_{th}$ is given by the 60th percentile computed on the *N* samples of $x_{p}$. The percentile value is fixed on the basis of an experimental analysis. The vector p is evaluated by comparing $x_{p}$ with $x_{th}$. In particular, it contains 1 when the corresponding sample of $x_{p}$ is higher than or equal to $x_{th}$; otherwise, it is 0:

(9) $$p\left(n\right)=\left\{\begin{array}{cc} 1, & \mathrm{if}\;x_{p}\left(n\right)\geq x_{th}\\ 0, & \mathrm{if}\;x_{p}\left(n\right)<x_{th} \end{array}\right.$$

where p(n) is the *n*-th element of the vector p, with n=1⋯,N. In this manner, the vector p presents ones where the ECG acquired in the selected leads has higher amplitude. Finally, the sensing matrix $\Phi \in {\left\{0,1\right\}}^{M\times N}$ is built by the vector p circularly shifting on each row by an amount equal to CR.

(10) $$\Phi =\left[\begin{array}{cccc} p(1) & p(2) & \cdots & p(N)\\ p(N-\mathrm{CR}+1) & p(N-\mathrm{CR}+2) & \cdots & p(N-\mathrm{CR})\\ \vdots & \vdots & \ddots & \vdots \\ p(\mathrm{CR}+1) & p(\mathrm{CR}+2) & \cdots & p(\mathrm{CR}) \end{array}\right].$$

The compression consists of the multiplication of the the vector $x_{l}$ acquired on each lead by the sensing matrix Φ, thus obtaining *M* compressed samples or overall the matrix $Y=\left[y_{1},y_{2},\cdots ,y_{L}\right]$ of size M×L.

(11) Y=ΦX.

The compression process can be interpreted as the cross-correlation of each vector acquired on the different leads with the p vector. The matrix Y and the vector p are then transmitted to the cloud server.

In the cloud server, the ECG received in compressed form can be reconstructed at the Nyquist rate. First of all, the sensing matrix Φ is rebuilt within the cloud server in the same manner as in the sensor node, thanks to the vector p. Moreover, the use of a sparsity matrix is required for the ECG reconstruction. In addition to the columns reported in (4), a further *N*-size column $u=\left(1/\sqrt{N}\right)\;\cdot \;{\left[1,\cdots ,1\right]}^{T}$ is employed to take into account a possible offset in the signal.

(12) $$\Psi =\left[\Psi^{\prime },u\right].$$

It is worth noting that the signals on the different leads are highly correlated, since they have the heart as common source and are synchronous. Therefore, they will share the same support, and the nonzero elements of each column $c_{\;\cdot \;l}$ will lie mostly in the same positions. This means that the matrix C is row-sparse; that is, it has few nonzero rows. Exploiting this consideration, the matrix X can be reconstructed by starting from the matrix of compressed samples Y (11), the dynamic sensing matrix Φ (10), and the Mexican hat wavelet matrix Ψ (12) and by solving a joint sparse recovery problem that can be expressed as the following [35]:

(13) $$\hat{C}=\arg\min_{C}{∥Y-\Phi \Psi C∥}_{F}^{2}+\lambda J,$$

where ${∥\;\cdot \;∥}_{F}$ denotes the Frobenius norm, λ is a regularization parameter that must be chosen to balance quality in the estimation of the cost function with sparsity [35,36], and *J* is a cost function that uses a *p*-norm, with p∈[0,1], in order to lead towards a sparse solution:

(14) $$J=\sum_{n=1}^{N}{∥c_{n\;\cdot \;}∥}_{2}^{p},$$

with $c_{n\;\cdot \;}$ the *n*-th row of C. The cost function (14) counts the number of non-null rows in C, and it is the extension to matrices of the $\ell_{0}$-norm that counts the non-null elements in a vector [35,36]. Once the $\hat{C}$ has been obtained, the matrix $\hat{X}$ is evaluated as follows.

(15) $$\hat{X}=\Psi \;\cdot \;\hat{C}.$$

The reconstruction is performed on each frame of M×L transmitted samples that are compressed in the sensor node from the N×L acquired samples. Fundamentally, through the proposed method, only the M×L matrix Y of the compressed samples is transmitted together with the *N*-size vector p in place of the N×L matrix X of the samples acquired at the Nyquist rate. The main advantage of the proposed method is the dynamic ECG evaluation. In other CS implementations for ECG monitoring, the sensing matrix Φ is usually randomly constructed according to a probability distribution [9,13,21,25,29]. As an example, in [21], the reconstruction performance of different CS methods is compared by considering several distributions, such as Bernoulli or Gaussian. However, adopting random matrices entails that the reconstruction performance may significantly vary depending on the correlation between the entries of the sensing matrix and the acquired samples. Such limitation is overcome in the proposed ECG monitoring system, since the sensing matrix is not randomly generated. Deterministic matrices have been already proposed in [22,23] as a viable alternative for ECG compression that does not require random number generation on the chip [22]. Indeed, random number generation on chip can be a heavy computational load for sensor nodes equipped with simple microcontrollers, while deterministic matrices could easily be employed also in wearable devices. The substantial difference of the proposed method with the other deterministic methods [22,23] is that the dynamic sensing matrix is built as a circulant matrix that depends on a combination of the leads with the most significant contribution. Thus, being adapted to the distribution of the acquired ECG, it contains more information on the signal features, guaranteeing better reconstruction quality. Finally, it is important to point out that the proposed method exploits the common information content that all the multi-lead signals share. In fact, only one sensing matrix and one sparsity matrix are adopted regardless the number of leads.

## 4. Implementation of the Proposed Method

The implementation of the proposed method for multi-lead ECG monitoring is presented in this Section. Both the phases of compression in the sensor node and reconstruction in the cloud server, described in previous Section 3, were implemented in the MATLAB environment. ECG signals from the Physikalisch-Technische Bundesanstalt (PTB) Diagnostic ECG Database, available online at the PhysioNet website [37], were examined. For each monitored patient, the signals related to the monitoring of 12 leads are available. For reducing the signal distortions due to the power line disturbances, the signals were filtered by a notch filter at the harmonics $f_{h}=h\;\cdot \;f$, with f= 50 Hz and h=1,2,⋯,9. Each signal of the PTB database is sampled at 1 k Samples/s. The duration of the signal frame was chosen equal to 1 s; therefore, the size of each frame results in N=1000. The size of *M* depends, instead, on the adopted CR.

When proposing a compression method, a good practice consists in verifying that the compression does not alter significantly the clinical information contained in the signal. The performance of a compression method for ECG signals and other biosignals is typically evaluated by the percentage of root-mean-squared difference (PRD) [9,12,13,21,23,24,25,26,28,29]. In this paper, the PRD is computed for the ECG signal related to each lead *l*:

(16) $$\mathrm{PRD}=\frac{{∥x_{l}-{\hat{x}}_{l}∥}_{2}}{{∥x_{l}∥}_{2}}\times 100\%,$$

where $x_{l}$ is the vector of the ECG samples acquired at the Nyquist rate, and ${\hat{x}}_{l}$ is the reconstructed vector, with ${∥\;\cdot \;∥}_{2}$ indicating the $\ell_{2}$-norm. In ECG monitoring, clinical information contained in the original signal acquired at the Nyquist rate is considered generally preserved if the reconstructed signal exhibits a PRD lower than 9% [21]. Therefore, in analyzing the proposed method, particular attention will be paid to such value as upper bound for good reconstruction and monitoring.

**Figure 2:**
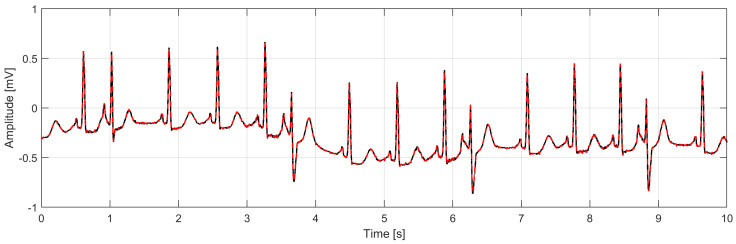
Lead I of the signal s0416 (myocardial infarction) from the PTB database for a time window of 10 s: the original frame is in black, and the frame reconstructed with CR=5 is in red.

**Figure 3:**
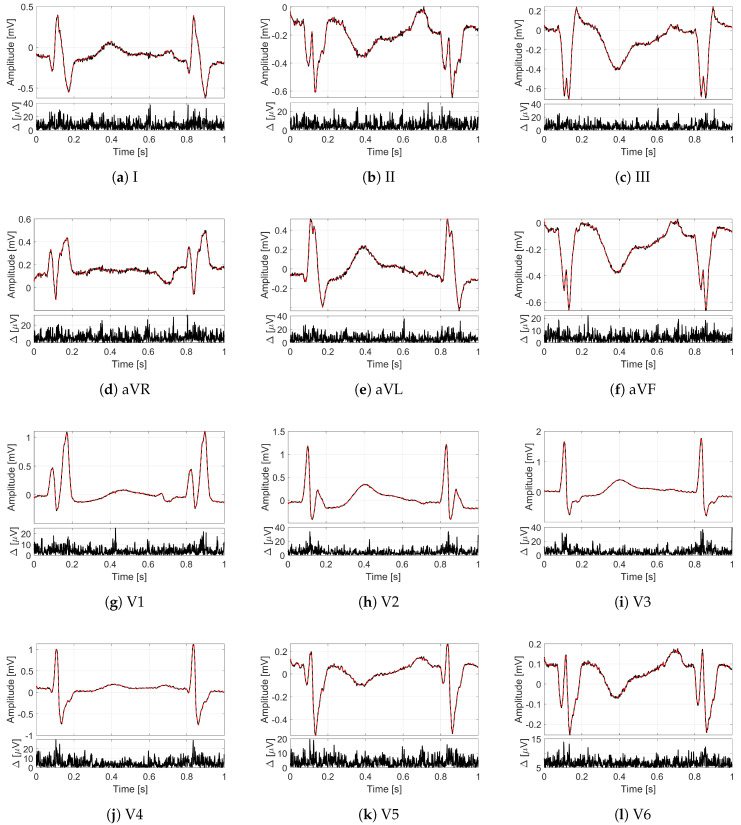
Twelve-leads ECG of the signal s0010 (myocardial infarction) from the PTB database for a time window of 1 s: (**a**) lead I, (**b**) lead II, (**c**) lead III, (**d**) lead aVR, (**e**) lead aVL, (**f**) lead aVF, (**g**) lead V1, (**h**) lead V2, (**i**) lead V3, (**j**) lead V4, (**k**) lead V5, (**l**) lead V6. On the top, the original frames are in black, and the frames reconstructed with CR=5 are in red; on the bottom, the absolute difference Δ between reconstructed and original frames.

In order to provide an idea of the reconstruction quality achieved by the proposed method for multi-lead ECG monitoring, Figure 2 and Figure 3 illustrate some recordings from the PTB database labeled as myocardial infarction. The minimization problem (13) with 12 measurement vectors was solved by using the M-FOCUSS algorithm [35], with regularization parameters fixed to $3\times 10^{-3}$. The original signals are drawn with black lines, while the signals reconstructed from M=200 compressed samples (i.e., with CR=5) are represented by red dashed lines. In particular, in Figure 2, the lead I of the signal s0416 is shown for a time window of 10 s where different deflections of the QRS complex can be appreciated. Figure 3 depicts, instead, all of the 12 leads of the signal s0010 for a time window of 1 s corresponding to a single frame. Moreover, for each lead, the absolute value of the difference Δ between original and reconstructed signals is reported too. What is worth noting is that all the 12 leads are correctly reconstructed. In fact, a good overlapping with the original signals can be appreciated not only for leads II and aVF (Figure 3b,f), which are employed to build the sensing matrix but also for all the other leads. The performance in terms of PRD confirms the graphical results since the obtained value for the signal of Figure 2 is 1.09%, while the obtained values for the signal of Figure 3 vary from a minimum of 1.99% for lead V2 to a maximum of 5.08% for lead I. In any case, the PRD for CR=5 is much lower than the limit of 9%.

## 5. Experimental Results

This section illustrates the performance of the proposed method for multi-lead reconstruction. The performance was evaluated in terms of PRD by several investigations on wide sets of signals from PTB database. The assessment was carried out in the MATLAB environment.

### 5.1. Analysis of Regularization Parameter

In order to compare the proposed method with the method [29], the multi-lead ECG monitoring based on the proposed dynamic method was initially implemented on the same signal set of [29]. The considered set comprises the following 10 signals labeled as myocardial infarction, either from men or women, with at least 60 years.

(17) $$S_{1}\;=\;\left\{s0010;s0026;s0035;s0037;s0039;s0045;s0047;s0052;s0053;s0056\right\}.$$

As a first step, the proposed method was implemented by considering two minimization algorithms to solve the reconstruction problem (13) with 12 measurement vectors: the Multiple Sparse Bayesian Learning (M-SBL) and the Multiple FOCal Underdetermined System Solver (M-FOCUSS) [35,36]. The two algorithms were evaluated depending on the regularization parameter λ for a time window of 10 s in terms of PRD and execution time. In more detail, for an overall performance evaluation, the PRD computed according to (16) for each lead of each signal of $S_{1}$ (17) was averaged over the 10 signals and the 12 leads. On the other hand, in order to take into account the actual duration employed by the two algorithms for the reconstruction of all the 12 leads simultaneously, the execution time was averaged over the frames of *N* samples related to $S_{1}$. This analysis was carried out for a time window of 10 s by setting CR=2.

**Figure 4:**
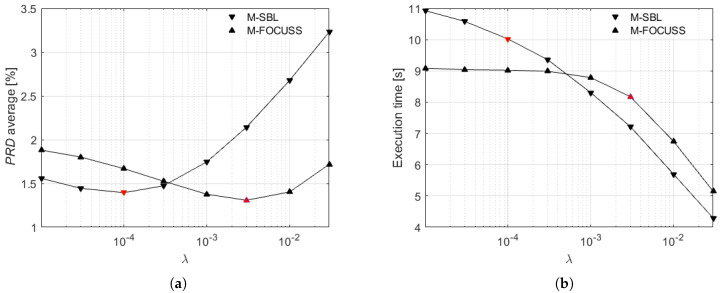
(**a**) Average PRD and (**b**) execution time of M-SBL and M-FOCUSS algorithms versus the regularization parameter λ, computed on the signal set $S_{1}$.

The experimental results of the two algorithms are presented in Figure 4. The PRD average, shown in Figure 4a, exhibits two different minimum values (marked in red) on the basis of the considered algorithm. In particular, the minimum value obtained by M-SBL algorithm is 1.39% corresponding to $\lambda =10^{-4}$, while the minimum value obtained by M-FOCUSS algorithm is equal to 1.31% for $\lambda =3\times 10^{-3}$. The execution time, shown in Figure 4b, presents instead for both the algorithms a decreasing trend as the λ values increase. Specifically, the execution times corresponding to the λ values related to the minimum PRD values are 10.02 s and 8.17 s for M-SBL and M-FOCUSS, respectively (as marked in red in Figure 4b). According to [28], the temporal M-FOCUSS algorithm [38] was analyzed too. However, this algorithm exhibited a PRD trend similar to M-FOCUSS but with a higher minimum PRD value and an execution time higher than 40 s. It is certain that M-FOCUSS not only provides the absolute minimum of PRD average but also the minimum execution time among the analyzed algorithms. Therefore, hereinafter, the adopted algorithm is only M-FOCUSS with the regularization parameter fixed to $3\times 10^{-3}$.

### 5.2. Performance Analysis

The performance of the proposed method was analyzed versus the number of leads as well as the compression ratio. To this aim, at first, the PRD was computed according to (16) for each considered lead; secondly, the PRD values related to the leads with the same label were averaged. Finally, average and standard deviations were evaluated over all the PRD values related to the different leads. For the results referring to a single lead, the average reports the test results, while the standard deviation is not reported.

The first investigation is intended to evaluate the performance of the proposed method as the number of leads employed by the ECG monitoring system increases. Specifically, the ECG monitoring system was considered composed by a number of leads L={1,3,6,8,10,12}. The analysis was conducted on the signal set $S_{1}$ (17) for a time window of 30 s.

**Figure 5:**
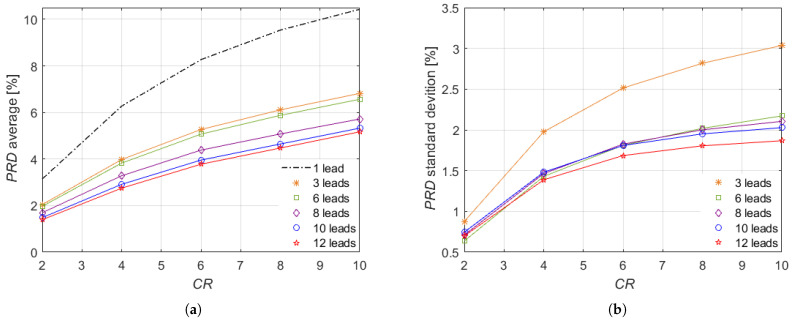
(**a**) Average and (**b**) standard deviation of PRD versus CR for the set of signals $S_{1}$ (myocardial infarction).

The results of this investigation are presented in Figure 5, depending on CR={2,4,6,8,10}. As expected, the average (Figure 5a) and the standard deviation (Figure 5b) increase with the CR values. It is worth noting that a significant reduction in the average PRD is achieved when multiple leads are used compared with the single lead case, thus demonstrating the advantage of a joint reconstruction from multiple leads. Such reduction increases with the number of leads. The best performance, both as average and standard deviation, is achieved by adopting all the conventional 12 leads. Then, by analyzing the average, it can be observed that 10 leads guarantee a trend similar to 12 leads. The performance decreases gradually by reducing the number of leads. Instead, as concerning the standard deviation, the results of 6, 8, and 10 leads are comparable. Therefore, if the higher number of measurement vectors is more difficult and heavier with respect to matching on the one hand, then on the other hand it adds further information on the coefficients of the ECG signal. According to the obtained results, the proposed approach generally exhibits an excellent performance for multi-lead ECG signal recovery. In fact, the results show a very low PRD up to CR=6, reaching CR=10 maximum values comprised between (5.17±1.87)% and (6.57±2.17)%, which is lower than the bound of 9% that is considered acceptable in medical applications [21].

**Figure 6:**
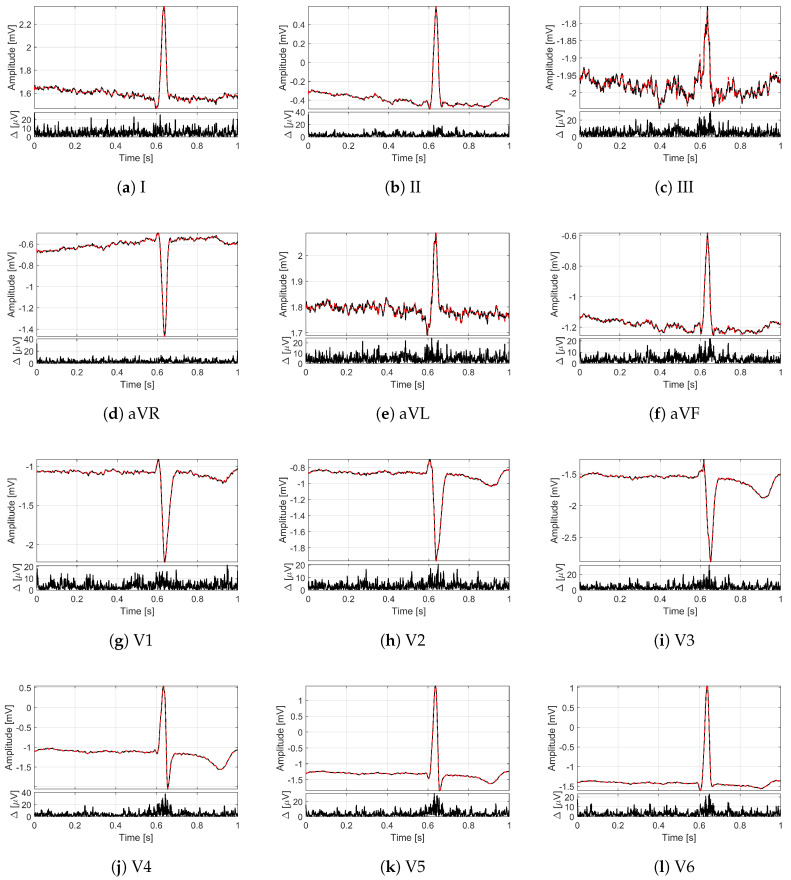
Twelve-leads ECG of the signal s0488 (cardiomyopathy) from the PTB database for a time window of 1 s: (**a**) lead I, (**b**) lead II, (**c**) lead III, (**d**) lead aVR, (**e**) lead aVL, (**f**) lead aVF, (**g**) lead V1, (**h**) lead V2, (**i**) lead V3, (**j**) lead V4, (**k**) lead V5, (**l**) lead V6. On the top, the original frames are in black, and the frames reconstructed with CR=5 are in red; on the bottom, the absolute difference Δ between reconstructed and original frames.

**Figure 7:**
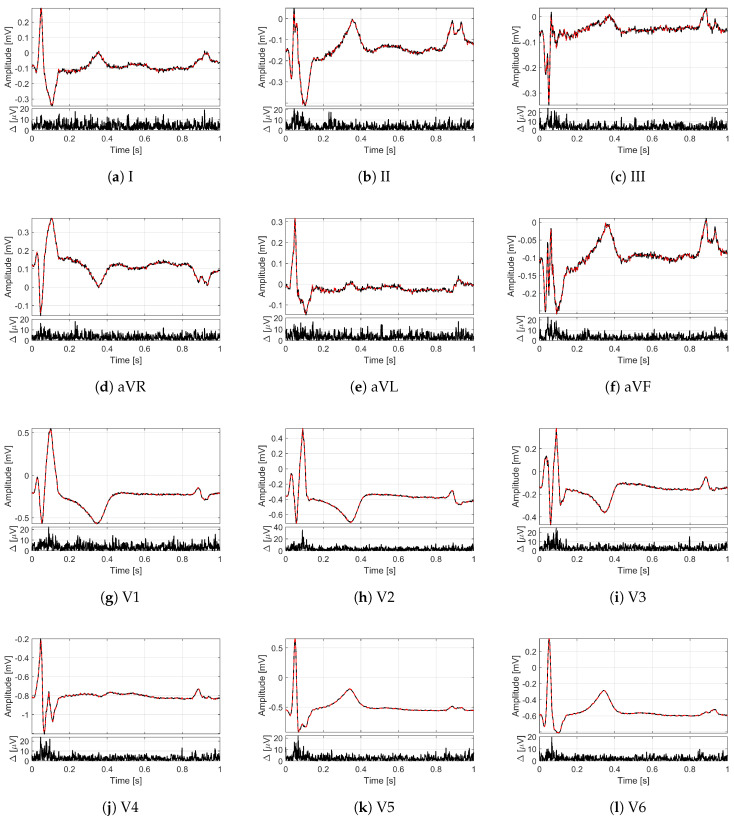
Twelve-leads ECG of the signal s0431 (bundle branch block) from the PTB database for a time window of 1 s: (**a**) lead I, (**b**) lead II, (**c**) lead III, (**d**) lead aVR, (**e**) lead aVL, (**f**) lead aVF, (**g**) lead V1, (**h**) lead V2, (**i**) lead V3, (**j**) lead V4, (**k**) lead V5, (**l**) lead V6. On the top, the original frames are in black, and the frames reconstructed with CR=5 are in red; on the bottom the absolute difference Δ between reconstructed and original frames.

Finally, for a more extensive examination of the performance of the proposed method, the PRD was also investigated on other sets of signals. In particular, since the previous assessments were carried out only on set $S_{1}$, characterized by all signals labeled in the PTB database as myocardial infarction, a further analysis was conducted on signals identified by different labels. Hence, the following sets of 10 signals were also considered:

(18) $$S_{2}\;=\;\left\{s0436;s0468;s0470;s0471;s0472;s0473;s0474;s0496;s0503;s0551\right\},$$

(19) $$S_{3}\;=\;\left\{s0383;s0423;s0437;s0444;s0456;s0488;s0489;s0492;s0493;s0498\right\},$$

(20) $$S_{4}\;=\;\left\{s0421;s0424;s0430;s0431;s0435;s0439;s0441;s0442;s0448;s0451\right\},$$

(21) $$S_{5}\;=\;\left\{s0002;s0340;s0432;s0484;s0485;s0494;s0508;s0509;s0510;s0546\right\},$$

where $S_{2}$ consists of signals acquired on people not suffering from any pathology and labeled as healthy controls, while $S_{3}$, $S_{4}$ and $S_{5}$ are sets of signals labeled by cardiac pathologies. In particular, $S_{3}$ and $S_{4}$ comprise signals labeled, respectively, as cardiomyopathy and bundle branch block. The reconstruction of all the 12 leads is reported in Figure 6 in the case of cardiomyopathy and in Figure 7 in the case of bundle branch block. $S_{5}$ includes, instead, several other pathologies: unstable angina, stable angina, myocarditis, hypertrophy, and dysrhythmia. The conventional number of 12 leads was chosen to evaluate the method in its full configuration. Moreover, in this case, the analysis was carried out for a time window of 30 s.

**Figure 8:**
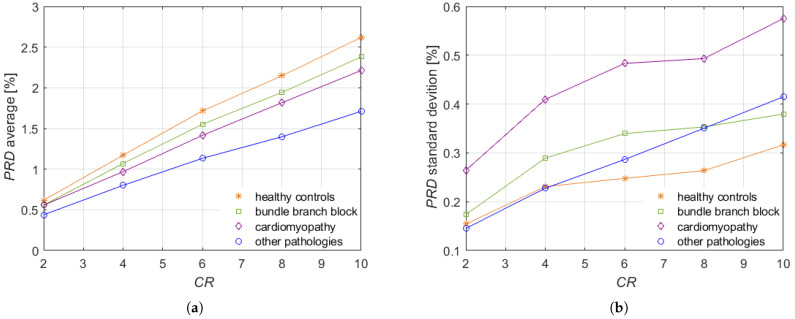
(**a**) Average and (**b**) standard deviation of PRD versus CR for sets of signals $S_{2}$, $S_{3}$, $S_{4}$ and $S_{5}$.

Figure 8 illustrates the obtained PRD values for CR={2,4,6,8,10}. The PRDs are even smaller than the ones obtained for set $S_{1}$. In particular, the average, in Figure 8a, is characterized by a linear trend with CR. From the analysis of Figure 8b, it can be noted that the standard deviations are lower than 1% for all the considered CR, so PRD values do not significantly deviate from the averaged values. Thus, the proposed method is capable of reconstructing well not only ECG signals related to healthy subjects but also to subjects affected by specific pathologies of cardiomyopathy and bundle branch block. In fact, for these two sets of signals, the proposed method provides maximum PRD values for CR=10 equal to (2.21±0.58)% and (2.38±0.38)%, respectively.

### 5.3. Comparison with Literature Methods

The proposed method was compared with the methods [7,29,30,31], all applying Compressed Sensing on multi-lead ECG signals and using the same signal database considered in this work. A first analysis has been carried out by comparing the results of the proposed method with those of the method [29]. In this case, the first 10 leads of the $S_{1}$ (17) dataset have been considered, and the average PRDs for each lead have been evaluated. The PRD was firstly computed lead by lead according to (16) for each signal of the set for a time window of 30 s. Then, the average of the PRD values related to the leads with the same label was evaluated.

Table 1 summarizes the results for CR={4,8} obtained by the proposed method and the method in [29]. As reported in the table, better results can be observed in the case of the proposed method for all the leads and for both the considered CR values. A further improvement can be appreciated also by comparing these results to the ones obtained by applying a preliminary version of the proposed method presented in [28]. The best result is achieved for lead I, and more generally, the values obtained through the proposed method are half of the values obtained by the random approach. It should be underlined that the bound of 9% is not reached for any of the leads, which is different from [29] where such bound is exceeded for most of the leads when CR=8 and for lead I in both of the considered CRs.

**Figure 9:**
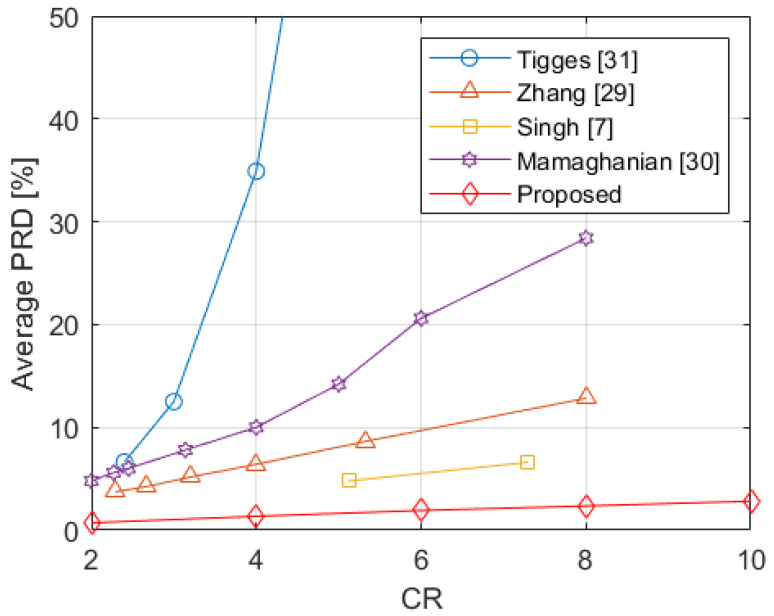
Comparison of the average PRDs obtained by the proposed method with the results reported in [7,29,30,31].

In Figure 9, the average PRD obtained with the application of the proposed method on all the considered datasets $S_{1},S_{2},S_{3},S_{4}$, and $S_{5}$, for different values of CR, is depicted. The figure also shows the results provided in [7,29,30,31]. In particular, for [31], the corresponding line reports the average along the considered 12 leads of the PRDs provided in Table 1 of that paper. For [30], the corresponding line reports the median values shown in Figure 3 (bottom) of the paper. The line referring to [29] reports the average of the PRD values along the 10 leads provided in Table I of [29] for model WL12M. In the case of [7], the corresponding line draws the average along the considered 12 leads of the PRDs reported in Tables 2 and 3 for the PTB database. Among the considered methods, the one presented in [31] achieves the highest values of average PRD, reaching values higher than 10% for a CR=3 and showing even higher values for increasing CRs. A slightly better performance is achieved by [30], where the average PRD values range from about 4.8% for CR=2 to about 28.4% for CR=8. The method in [29] shows values increasing almost linearly from about 3.7% for CR=2.29 up to about 12.8% for CR=8. The method in [7] further improves reaching an average PRD of about 4.8% for a CR=5.12 and 6.6% for a CR=7.3. The proposed method achieves the best results versus the considered methods and results in a curve that lies below all the others, ranging almost linearly from 0.71% for CR=2, to 2.82% for CR=10.

## 6. Conclusions

In this paper, a CS-based method for multi-lead ECG signal monitoring has been presented. In detail, the proposed method employs a deterministic sensing matrix dynamically built from a vector obtained by a proper combination of ECG signals of two different leads. According to such vectors, for each ECG frame, a compressed version of the signal is obtained and then transmitted to the cloud server by the sensor node, together with the vector determining the sensing matrix. Thus, in the cloud server, the sensing matrix can be rebuilt, and all the ECG leads can be recovered. Specifically, the sparsity matrix is based on a Mexican hat wavelet kernel.

The method was evaluated through several investigations on a wide set of signals. The PRD values obtained from the proposed method were analyzed against the number of considered leads and CR. The experimental results show better performance in case of ECG monitoring system with all the conventional 12 leads. In any case, the PRD values are always lower than the bound of 9%, which has been indicated for the preservation of ECG information up to CR=10 and reveals the proposed method suitability for ECG monitoring of subjects considered healthy as well as affected by pathologies such as myocardial infarction, cardiomyopathy, and bundle branch block. Furthermore, the method was compared with other four relevant literature papers proposing CS for multi-lead ECG monitoring. The proposed method improves significantly the signal reconstruction quality, as demonstrated by the lowest PRD obtained in the experimental results among all the considered CS methods.

As future work, the implementations and testing on the hardware of the proposed method have been planned with the aim of demonstrating its suitability to be implemented on wearable devices in IoMT applications.

## Figures and Tables

**Table 1:** PRD values for the set of signals $S_{1}$ in comparison to the values obtained in [29].

CR	Multi-Lead Reconstruction	PRD (%)									
		I	II	III	aVR	aVL	aVF	V1	V2	V3	V4
4	Zhang [29]	11.95	5.96	6.81	8.09	9.23	6.31	4.69	3.18	4.11	3.71
	proposed method	6.10	2.75	2.74	3.91	4.39	2.38	2.23	1.50	1.52	1.61
8	Zhang [29]	21.68	10.91	13.05	14.89	17.01	12.07	9.69	7.76	10.97	10.26
	proposed method	8.97	4.36	4.29	5.92	6.50	3.87	3.74	2.70	2.95	3.15

## Data Availability

No new data were created in this study. Data sharing is not applicable to this article.
